# Towards the Use of Metabolic Volatiles in Breath for Determining Drug Response: Gstachamine as an Unlabeled Substrate to Measure CYP3A4 Activity

**DOI:** 10.1002/cmdc.202500492

**Published:** 2025-08-25

**Authors:** Valentina Stock, Rebecca Hofer, Klaus R. Liedl, Jakob Troppmair, Thierry Langer, Hubert Gstach, Christian Dank, Sarah Kammerer, Veronika Ruzsanyi

**Affiliations:** ^1^ Institute for Breath Research University of Innsbruck Innrain 80/82 6020 Innsbruck Austria; ^2^ Department of General Inorganic and Theoretical Chemistry University of Innsbruck Innrain 80/82 6020 Innsbruck Austria; ^3^ Daniel Swarovski Research Laboratory Department of Visceral Transplant and Thoracic Surgery Medical University of Innsbruck Innrain 80/82 6020 Innsbruck Austria; ^4^ Department of Pharmaceutical Chemistry University of Vienna Althanstraße 14 1090 Vienna Austria; ^5^ Institute of Organic Chemistry University of Vienna Währinger Straße 38 1090 Vienna Austria; ^6^ Institute of Biotechnology Molecular Cell Biology Brandenburg University of Technology Cottbus‐Senftenberg Universitätsplatz 1 01968 Senftenberg Germany

**Keywords:** biotransformation, butanone, CYP3A4, mass spectrometry, volatile metabolites

## Abstract

Breath analysis is a promising noninvasive diagnostic tool, but the clinical applicability of breath tests depends on several factors. A salient criterion pertains to the presence of substrates with the ability to produce detectable volatile metabolites during the metabolism. In this work, we evaluated the potential of two candidate compounds, namely gstachidine and gstachamine, for their use in metabolic breath analysis. Both substrates were evaluated for their toxicity and metabolic conversion in HepG2 cell clones overexpressing CYP3A4. Gstachidine was found to be toxic and did not produce any volatile metabolite. In contrast, gstachamine successfully generated butanone as a volatile metabolite, making it the first substrate to yield a stable VOC detectable exclusively at low ppb_V_ levels in breath. To characterize its biotransformation, we conducted time‐dependent analyses, as well as CYP specificity, toxicity, and inhibition investigations regarding the production of *N*‐dealkylated gstachamine or butanone. The results demonstrated that gstachamine had a high metabolic turnover and a strong CYP3A4‐dependency in the production of the specific *N*‐dealkylated metabolite. Furthermore, a substantial reduction in the production of both metabolites was observed in HepG2‐CYP3A4 cells following treatment with CYP inhibitors 1‐aminobenzotriazole or ketoconazole. The results suggest that gstachamine has potential for noninvasive CYP3A4 metabolism monitoring.

## Graphical abstract

A potential substrate, gstachamine, was engineered for a future CYP3A4 assay. HepG2 cell clones overexpressing CYP3A4 were used to investigate both liquid and headspace phases after incubation. A specific *N*‐dealkylated metabolite and butanone were detected using LC‐MS and PTR‐ToF‐MS, respectively. As a volatile compound found in the low ng L^−1^ levels in breath, butanone supports gstachamine's potential for CYP3A4‐specific breath testing.

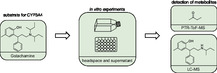

## 1 Introduction

Personalized drug therapy is predicated on the ability to predict the metabolism of each patient, given that individual variations of the patients in metabolic processes influence the drug efficiency. A key factor contributing to this inter‐individual variability is the activity of cytochrome P450 (CYP) enzymes, which play a pivotal role in the metabolism of wide range of xenobiotics.^[^ ^1^, ^2^, ^3^ ^]^ Due to the polymorphisms of CYP genes, patients can be of four different metabolic phenotypes, namely ultrarapid, extensive, intermediate, and poor metabolisers.^[^ ^4^ ^]^ Theses polymorphisms, in conjunction with other factors such as environmental interactions, have the potential to result in adverse drug effects and altered clinical drug responses.^[^ ^5^ ^]^

Noninvasive breath tests have become a valuable tool for assessing metabolic function and enzyme activity. By analyzing exhaled volatile compounds—products of enzymatic reactions in the body—these tests can offer insight into metabolic processes.^[^ ^6,^, ^7^ ^]^ Breath analysis, particularly ^13^C‐breath tests, provide a noninvasive, simple, and safe diagnostic approach allowing rapid screening and continuous monitoring. In addition, such tests may serve as companion diagnostics to determine the CYP phenotype.^[^ ^8^ ^]^

Despite the numerous advantages of these noninvasive tests, they are not without limitations. For instance, the majority of these tests are based on isotopically labeled CO_2_, which increases costs due to the labeling process in their manufacturing. Additionally, the baseline is relatively high, with approximately 5% CO_2_ already present in the exhaled breath of every patient and hence approximately 500 ppm_V_^13^CO_2_. Therefore, the metabolite must be generated in concentrations well above the baseline, which in turn demands a comparatively high quantity of precursor compound. Consequently, there is a need to explore alternative markers yielding an unlabeled, volatile metabolite which is either absent or present only at very low concentrations (in the ng L^−1^ range) in exhaled air. Initial own experiments with tolterodine and diisopromine revealed that acetone was produced as a volatile metabolite following their biotransformation by CYP3A4.^[^ ^9,^, ^10^ ^]^ However, acetone is not an ideal candidate as it is already present in around 300–1000 ppb_V_ in adults’ breath,^[^ ^11^ ^]^ albeit at a much lower baseline compared to CO_2_.

In designing new precursors, several factors must be considered, including solubility, the presence or absence of a stereocenter and the stability of the resulting metabolites. The chemical structure of a pharmaceutical compound plays a crucial role for modifying and optimizing its pharmacokinetical properties. Chemical modifications of substrates and drugs—such as the removal of a hydroxyl and a methyl group—have been shown to enhance biotransformation efficiency and to decrease toxicity.^[^ ^10^ ^]^

**Figure 1:**
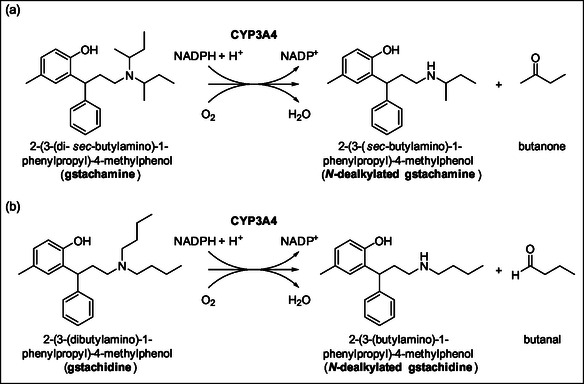
Proposed metabolic pathway of a) gstachamine and b) gstachidine by the CYP3A4 enzymes to a nonvolatile *N*‐dealkylated metabolite as well as a volatile metabolite butanone or butanal, respectively.

A purposeful chemical modification of precursors could potentially lead to the formation of volatile metabolites that are not typically present in exhaled air. Accordingly, the present study focused on the biotransformation of two modified variants of tolterodine. In both cases, the isopropyl groups on the side chain of the *N*‐moiety were changed by C4‐groups. In the first derivate a di‐*sec*‐butyl was attached to the *N*‐moiety (see **Figure** **1**a), resulting in 2‐(3‐(di‐*sec*‐butylamino)‐1‐phenylpropyl)‐4‐methylphenol referred to as gstachamine. In the second substrate, 2‐(3‐(dibutylamino)‐1‐phenylpropyl)‐4‐methylphenol (gstachidine), a di‐*n*‐butyl group was introduced instead (Figure 1b).

Given the structural similarity of these two substrates to tolterodine, it was assumed that both compounds can undergo *N*‐dealkylation by CYP3A4. In each case, we expected the production of a specific nonvolatile *N*‐dealkylated metabolite along with a volatile metabolite, namely a) a ketone (butanone) from gstachamine and b) an aldehyde (butanal) from gstachidine. As these are entirely novel chemical entities, no data currently exist regarding their biotransformation pathways, including the CYP enzymes involved and the resulting metabolic products. Both butanal and butanone are typically present at low levels (below 10 ppb_V_) in exhaled air.^[^ ^12^, ^13^, ^14^ ^]^ Due to their low baseline concentrations in the breath, any metabolic‐induced changes during a potential breath test would be more easily distinguishable, thereby offering greater sensitivity and specificity as biomarkers.

The present work aimed to investigate two modified versions of tolterodine as possible CYP3A4 substrates that were expected to produce butanone and butanal. To this end, we evaluated their CYP‐mediated metabolism in genetically modified human hepatoblastoma‐derived HepG2 cells, conducted toxicity assessments, performed time‐dependent analyses, and investigated enzyme inhibition effects. Apart from the volatile metabolite production, the formation of the more specific *N*‐dealkylated metabolite was also investigated. These findings contribute to a more comprehensive understanding of underlying metabolic pathways and support the development of novel breath precursors for diagnostic applications.

## 2 Results and Discussion

### 2.1 Biotransformation of Gstachamine and Gstachidine

**Figure 2:**
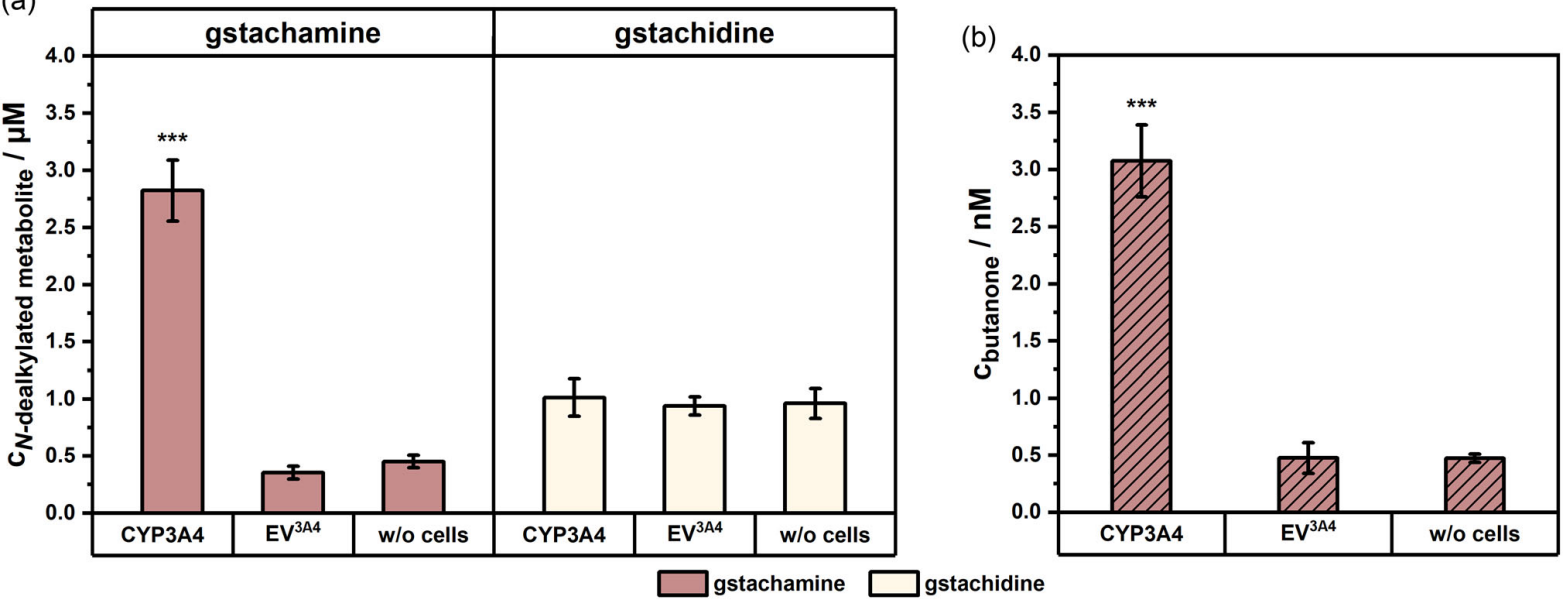
Comparison of the two substrate candidates gstachamine and gstachidine after 260 min biotransformation with 110 µM of the respective substrate. In a), the production of the nonvolatile *N*‐dealkylated metabolite as measured by LC‐MS and in b) the production of the volatile metabolite butanone after gstachamine biotransformation as measured by PTR‐ToF‐MS. In addition, the nonenzymatic degradation of the substrate (w/o cells) is shown, in which the T75 flasks were treated in the same way as the cells, except that no cells were cultured in them. Groups were compared using one‐way ANOVA followed by Tukey's multiple comparisons test. Statistical significance at *p* < 0.001 (***) relative to w/o cells (*n* ≥ 2 independent experiments, each performed with at least technical duplicates).

To confirm the proposed biotransformation pathway illustrated in Figure 1 for gstachamine and gstachidine, compounds were incubated with the CYP3A4 reporter cell lines. During biotransformation, the formation of a substrate‐specific *N*‐dealkylated metabolite was predicted in the liquid phase surrounding the cells attached to the bottom of the tissue culture flask, while the volatile metabolites butanone (from gstachamine) and butanal (from gstachidine) were expected in the headspace. After a reaction time of 260 min, the production of the nonvolatile metabolites is shown in **Figure** **2**a. As anticipated, the biotransformation of gstachamine by HepG2‐CYP3A4 cells exhibited a high metabolic turnover, resulting in the production of 2.82 ± 0.27 µM *N*‐dealkylated gstachamine. In contrast, only 0.36 ± 0.06 µM of the metabolite was formed in HepG2‐CYP3A4 empty vector (EV) cells. The nonenzymatic degradation of gstachamine accounted for 0.45 ± 0.05 µM as seen in the incubation condition without any cells (‘w/o cells’).

Conversely, approximately 1 µM *N*‐dealkylated gstachidine was detected in both HepG2‐CYP3A4 and EV cells following incubation with gstachidine (Figure 2a). Since the nonenzymatic degradation of gstachidine also yields a concentration of approximately 1 µM, it can be assumed that no biotransformation has occurred. Furthermore, it was observed that the cells gradually died following exposure to gstachidine after the given incubation time, hence, it can be assumed that the chemical structure of gstachidine is sufficiently toxic to the cells.

The production of the volatile metabolite butanone after biotransformation with gstachamine is shown in Figure 2b. The presence of gstachamine resulted in the formation of 3.08 ± 0.31 nM butanone in CYP3A4‐overexpressing cells. The concentrations detected in the EV control cells and the nonenzymatic degradation of gstachamine were above the LOD (0.14 nM) and around the LOQ (0.49 nM) of the PTR‐ToF‐MS measurements.

In contrast, no production of butanal was observed following exposure to gstachidine, supporting the hypothesis that no biotransformation occurred, likely due to the toxic nature of gstachidine. One possible explanation is the differing chemical stability of the volatile metabolites: while butanone, a ketone, is relatively stable, aldehydes such as butanal are generally more reactive and can be rapidly metabolized in the human body, for instance to 1‐butanol or butyric acid.^[^ ^15^ ^]^ This rapid conversion is likely due to the body's need to eliminate reactive compounds quickly, leading to their oxidation or reduction to less reactive and more easily excretable compounds. Based on these findings, particularly the absence of any volatile metabolite and its toxicity, gstachidine was excluded as a potential candidate for breath testing. Subsequent investigations focused solely on gstachamine.

### 2.2 Time‐Dependent Biotransformation of Gstachamine

**Figure 3:**
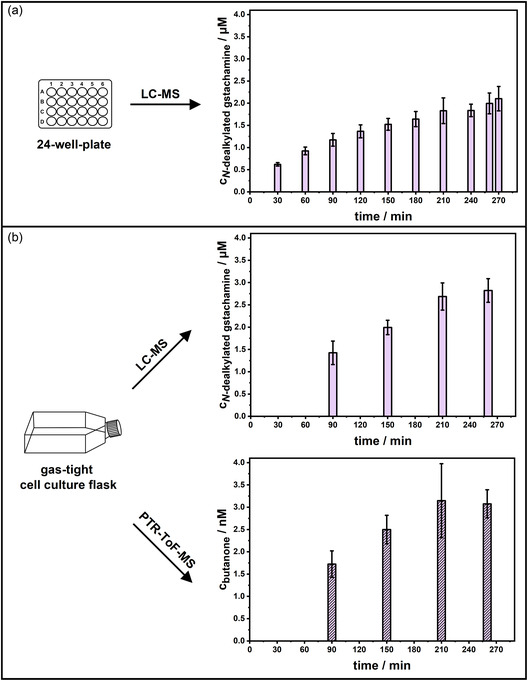
Time‐dependent production of *N*‐dealkylated gstachamine and butanone by HepG2‐CYP3A4 cells treated with 110 µM gstachamine a) over a period of 270 min in a 24‐well‐plate and b) at four specified time points in a gas‐tight T75 cell culture flask. The supernatants and the headspace above the cell were sampled at specific time points and analyzed using LC‐MS and PTR‐ToF‐MS, respectively (*n* ≥ 2 independent experiments, each performed with at least technical duplicates).

The comparatively high concentrations of both volatile and liquid metabolites enable the reduction of the incubation time, as shortening the analysis time is essential for implementing a breath test. For this purpose, HepG2‐CYP3A4 cells were incubated with gstachamine in a 24‐well plate (**Figure** **3**a) for predefined time periods (30–260 min). The EV control values were disregarded in this setup as they were negligibly small compared to those of the CYP3A4 overexpressing cells, but above the LOQ (7.86 nM) and LOD (1.97 nM). The concentration of the *N*‐dealkylated gstachamine produced was observed to increase over time, ultimately slowing to a plateau after approximately 180–210 min.

The biotransformation of 110 µM gstachamine to *N*‐dealkylated gstachamine and butanone in cell culture flasks (with the attached sampling device) at specified time points (90 min, 150 min, 210 min, or 260 min) is presented in Figure 3b. The results for the *N*‐dealkylated gstachamine are slightly different from those in the 24‐well plate, as higher levels are obtained from the cell culture flask. After 260 min, 2.82 ± 0.27 µM *N*‐dealkylated gstachamine was formed, whereas in the 24‐well experiment, only 2.00 ± 0.23 µM was produced. The reason for this discrepancy could be explained by the different cell aqueous volume ratio and the scale of the experiment.

The production profile of the volatile metabolite butanone closely mirrors that of the nonvolatile metabolite. From 210 min onwards, a slight decline in metabolite levels became apparent. After 150 min of incubation, 1.99 ± 0.16 µM *N*‐dealkylated gstachamine and 2.50 ± 0.32 nM butanone were detected. The 1000‐fold difference between the concentrations of volatile and nonvolatile metabolites can be well explained by the equilibrium of the volatile metabolite between the gas and liquid phases.^[^ ^9^ ^]^ Since butanone is produced as a volatile metabolite from this new substrate, the ratio between the volatile metabolite concentration in the buffer and in the headspace was again determined. To this end, 2 µM butanone in KHB was added to the cell culture flask, corresponding to the amount of *N*‐dealkylated gstachamine after 150 min of incubation. The butanone concentration in the gaseous phase following equilibrium was determined to be 1.75 ± 0.44 nM, which is close to the range of the butanone concentrations measured in the cell culture headspace.

Given the observed decline in the production of both metabolites starting at 210 min, along with a notably high standard deviation in the PTR‐ToF‐MS measurement at the same time point, it was decided to conduct all subsequent experiments using an incubation time of 150 min.

### 2.3 Determining Gstachamine Conversion in Different CYP Enzymes Overexpressing HepG2 Cell Clones

**Figure 4:**
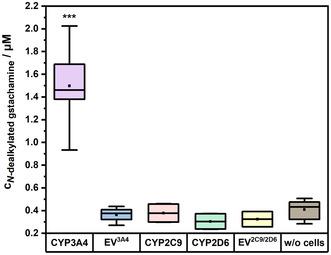
A comparative analysis of HepG2 cell clones overexpressing CYP3A4, CYP2C9, or CYP2D6, alongside their corresponding EV clones after an incubation with 110 µM gstachamine for 150 min monitoring the *N*‐dealkylated metabolite. For the nonenzymatic degradation of the substrate (w/o cells), wells were treated in the same way as the cells, except that no cells were cultured in the wells. Cell supernatant of the wells was collected and measured with LC‐MS. Groups were compared using one‐way ANOVA followed by Tukey's multiple comparisons test. Statistical significance at *p* < 0.001 (***) relative to w/o cells (*n* ≥ 2 independent experiments, each performed with at least technical duplicates). The data are presented as box plots, with the median indicated by a horizontal line, the interquartile range (IQR) shown as the colored box (25th–75th percentile), and the whiskers representing the minimum and maximum values within 1.5 times the IQR. The small square within each box denotes the mean.

To assess the substrate's specificity toward cytochrome P450 enzymes, three isoforms—CYP3A4, CYP2C9 and CYP2D6—were selected based on their established involvement in the biotransformation of tolterodine.^[^ ^16^ ^]^ **Figure** **4** illustrates the produced *N*‐dealkylated metabolite concentrations in µM by the tested isoforms. Notably, the CYP3A4 isoform generated significantly higher levels of *N*‐dealkylated gstachamine (1.50 ± 0.30 µM) compared to other isoforms. In contrast, CYP2C9 and CYP2D6 produced metabolite levels comparable to their respective EV controls at a concentration of about 0.4 µM. Moreover, the chemical degradation in the absence of cells (‘w/o cells’) was already elevated reaching 0.41 ± 0.08 µM. These results indicate that among the tested isoforms, only CYP3A4 has a substantial impact on the production of the *N*‐dealkylated metabolite.

**Figure 5:**
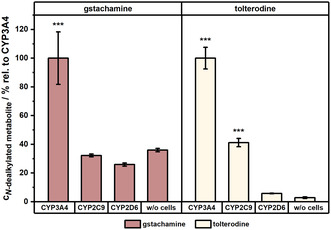
Relative production of the *N*‐dealkylated metabolite, normalized to CYP3A4 activity, in HepG2 cells overexpressing CYP3A4, CYP2C9, or CYP2D6 after treatment with either 110 µM gstachamine or tolterodine for 150 min. The nonenzymatic degradation of the substrate (w/o cells) was treated in the same way as the HepG2‐cell clones, except that no cells were cultured in the 24 well plate. Cell supernatants were collected and analyzed by LC‐MS. Groups were compared using one‐way ANOVA followed by Tukey's multiple comparisons test. Statistical significance at *p* <  0.001 (***) relative to w/o cells (*n* = 2 independent experiments, each performed with technical duplicates).

**Figure** **5** displays the relative production of the *N*‐dealkylated metabolite from gstachamine in comparison to tolterodine by HepG2 cells overexpressing the three CYP isoforms selected, expressed as percentage of the CYP3A4 isoform. While CYP2C9 appears to generate over 30% of the *N*‐dealkylated gstachamine relative to CYP3A4, this value falls within the range of the chemical degradation in the absence of cells (‘w/o cells’). In the case of tolterodine, the CYP2C9‐catalyzed *N*‐dealkylation resulted in the production of approximately 40% of the *N*‐dealkylated metabolite relative to CYP3A4, with a lower chemical degradation (less than 5%). Consequently, an extension of the side chains at the nitrogen atom of the tolterodine structure alters substrate recognition and, therefore affects CYP specificity.

### 2.4 Concentration‐Dependent Effects of Gstachamine

**Figure 6:**
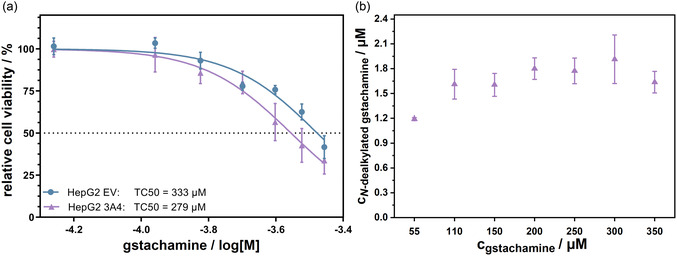
a) Concentration dependent determination of the relative cell viability after treatment (150 min) with varying concentrations of gstachamine up to 350 µM on HepG2‐CYP3A4‐ and EV cell clones using a trypan blue exclusion assay. b) Production of the *N*‐dealkylated gstachamine in µM after treatment with an increasing gstachamine concentration on HepG2‐CYP3A4 cell clones for 150 min. The cell supernatant was analyzed by LC‐MS (*n* ≥ 2 independent experiments, each performed with at least technical duplicates).

In order to investigate the concentration dependent effects of the substrate gstachamine, the cell viability (**Figure** **6**a) and the production of the nonvolatile metabolite (Figure 6b) were analyzed. At concentrations up to 150 µM, the relative cell viability was above 85% for both HepG2‐CYP3A4 and EV cells. Subsequently, cell viability decreased more rapidly, resulting in TC_50_ values of 279 µM and 333 µM for HepG2‐CYP3A4 and ‐EV, respectively. Given that the TC_50_ value of the HepG2‐CYP3A4 is only slightly lower, it can be assumed that CYP3A4 does not produce a toxic metabolite or that the metabolite exerts only a negligible influence on the relative cell viability. Looking at the amount of *N*‐dealkylated gstachamine produced, there is an increase from 55 µM to 110 µM, with all higher concentrations falling within the same range. As gstachamine became increasingly cytotoxic beyond 110 µM, the associated reduction in viable cell numbers likely accounted for the equally high levels of the nonvolatile metabolite at higher substrate concentrations.

A challenge in determining the TC_50_ values was the limited solubility of gstachamine in KHB at concentrations exceeding 400 µM, which may have affected the accuracy of the measured data in this range. Nevertheless, since the production of *N*‐dealkylated gstachamine reached a plateau at 110 µM, this concentration represents a reliable and practical choice for subsequent applications.

A comparison of the toxicity of gstachamine, tolterodine, and diisopromine showed that the latter two compounds have higher TC_50_ values than gstachamine in HepG2‐CYP3A4 cells (tolterodine 414 µM and diisopromine 959 µM).^[^ ^9,^, ^10^ ^]^ It is important to note that tolterodine and diisopromine each were incubated for 4 h, whereas gstachamine was incubated for 150 min. Interestingly, gstachamine and tolterodine exhibited relatively low TC_50_ values, while diisopromine reached nearly 1000 µM. This suggests that the *p*‐cresol structure, present in both tolterodine and gstachamine, may potentially contribute to their increased toxicity. Future studies could investigate whether the diphenyl variant of gstachamine exhibits similarly elevated TC_50_ values, providing additional insight into the relationship between structure and toxicity.

### 2.5 Inhibitory Effects of 1‐ABT and Ketoconazole on the Biotransformation of Gstachamine

**Figure 7:**
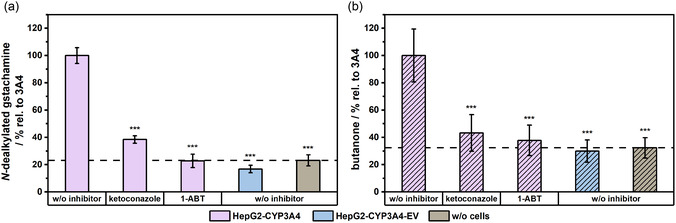
The influence of 1 mM 1‐ABT and 5 µM ketoconazole on the biotransformation of gstachamine within HepG2‐CYP3A4 and EV cells was assessed in relation to the production of a) *N*‐dealkylated metabolite and b) butanone after 150 min of incubation time with 110 µM gstachamine. The nonenzymatic degradation of the substrate (w/o cells) condition was treated in the same way as the cells, except that no cells were cultured in the T75 flasks. Cell supernatants and headspace were obtained and analyzed via LC‐MS and PTR‐ToF‐MS, respectively. Groups were compared using one‐way ANOVA followed by Tukey's multiple comparisons test. Statistical significance at *p* < 0.001 (***) relative to HepG2‐CYP3A4 cells subjected to treatment without the inhibitor (*n* ≥ 2 independent experiments, each performed with at least technical duplicates). The dashed line indicates the level of the nonenzymatic degradation (w/o cells).

To investigate the CYP3A4‐mediated inhibition of gstachamine, the effects of 1‐ABT (pan‐CYP inhibitor) and ketoconazole (specific CYP3A4 inhibitor) were examined by measuring the production of both the *N*‐dealkylated metabolite and the volatile metabolite butanone. As demonstrated in **Figure** **7**, a significant inhibition was observed in both metabolite productions, thereby confirming the involvement of CYP3A4 in the biotransformation process. Figure 7a illustrates that the production of the nonvolatile metabolite was significantly inhibited, with only 38.4 ± 2.7% and 22.8 ± 4.9% of the *N*‐dealkylated gstachamine produced following treatment with ketoconazole and 1‐ABT, respectively relative to untreated CYP3A4 overexpressing cells. The EV control yielded values consistent with the range of the nonenzymatic chemical degradation of the substance ('w/o cells'). In comparison, the inhibition of the headspace above the cells (Figure 7b) resulted in butanone levels of 43.2 ± 13.5% and 37.8 ± 11.2% relative to the CYP3A4 cells after ketoconazole and 1‐ABT treatment, respectively. The nonenzymatic degradation and the EV control resulted in values below 35%.

Overall, these results indicate that the production of both nonvolatile and volatile metabolites is inhibited by approximately 55–75% with the respective inhibitors. These findings are consistent with previous observations for tolterodine and diisopromine biotransformation,^[^ ^9,^, ^10^ ^]^ where strong inhibition was also detected. Differences were observed in the production of the nonvolatile metabolite. *N*‐dealkylated metabolite levels in case of tolterodine and diisopromine dropped to values below 20% after inhibition, particularly with ketoconazole. While slightly higher levels of the nonvolatile metabolite were observed in the gstachamine biotransformation, this is likely due to an increased nonenzymatic degradation of the substrate.

## 3 Conclusion

When comparing gstachamine with tolterodine and diisopromine—both of which generate acetone upon biotransformation and have been explored as alternatives to isotopically labeled CO_2_—gstachamine offers a significant advantage: it produces a volatile organic compound (VOC), butanone, which is typically present at only low baseline levels in exhaled breath. Given that only small quantities of substrate (typically a few hundred milligrams) are used in breath tests, the metabolic efficiency of gstachamine suggests that even minimal doses (in the milligram range) could yield detectable levels of butanone above baseline concentrations. Although some of the butanone may be further metabolized in the human body, a measurable portion is likely to be exhaled and thus detectable. Future studies should aim to determine the extent of butanone oxidation *in vitro* and *in vivo* *.*

In the present study, experiments were conducted using 110 µM gstachamine, revealing a substantial difference in metabolite production between CYP3A4‐overexpressing and EV cells. These results suggest that lower concentrations could be sufficient for future applications, potentially reducing both costs and the risk of side effects while enhancing clinical feasibility.

Further optimization of the gstachamine structure should be explored in future research to enhance its suitability for the use in breath assays. Previous findings^[^ ^10^ ^]^ have indicated that removing the hydroxyl and methyl groups from the *p*‐cresol moiety, thereby eliminating the stereocenter, may improve both metabolic turnover and the TC_50_ value. Additionally, modifying the *N*‐moiety may lead to the generation of novel, nonlabeled VOCs with unique metabolic signatures.

We note that this research constitutes a proof‐of‐principle investigation conducted at the cellular level. While HepG2‐CYP3A4 cells have proven valuable for investigating CYP3A4‐dependent metabolism of gstachamine, they do not fully capture the complexity of human physiology. Future studies should employ more physiologically relevant liver models, such as primary or primary‐like human hepatocytes, to better reflect human liver metabolism. Moreover, to assess toxicity and possible side effects *in vivo*, blood routine tests involving animal models should also be conducted. These advancements will support the transition to clinical studies evaluating the potential of gstachamine as a breath test candidate.

The transition to the clinical studies also depends on the availability of appropriate gas sensing devices for measuring the specific breath volatiles produced. In this regard, sensors represent a cost‐effective solution for a fast, on‐line detection of VOCs in exhaled air. Currently, several types of sensors, including chemoresistive sensors based on Si/WO_3_ nanoparticles or conducting polymers, are available for acetone monitoring.^[^ ^17,^, ^18^ ^]^ Furthermore, these gas sensors offer the advantage of being compact, hand‐held devices that can be operated without the need for scientifically trained personnel.

Despite the limitations of this study, it lays a strong foundation for continued research into CYP activity testing using gstachamine as a model substrate. These findings contribute to the broader development of noninvasive diagnostic assays capable of predicting individual drug responses through breath analysis.

## 4 Experimental Section

4.1

4.1.1

##### Reagents and Chemicals

Sodium hydrogencarbonate (>99.5%) was supplied by Merck KGaA (Darmstadt, Germany). Acetonitrile (ACN, ≥99.9%), butanone (>99.5%), calcium chloride dihydrate (≈99%), dimethyl sulfoxide (DMSO, ≥99.9%),4‐(2‐hydroxyethyl)piperazine‐1‐ethanesulfonic acid (HEPES, >99.5%), methanol (≥99.9%, HPLC grade), modified Krebs‐Henseleit‐Buffer (KHB), and trypan blue solution (0.4%) were purchased from Sigma Aldrich (St. Louis, MO, USA). 1‐Aminobenzotriazole (1‐ABT, >98.0%), ketoconazole (>98.0%), and tolterodine L‐tartrate (>98.0%) were acquired from Tokyo Chemical Industry (TCI chemicals, Tokyo, Japan). *N*‐dealkylated tolterodine (tolterodine impurity E CRS) was purchased from LGC standards (Wesel, Germany). Dulbecco's modified Eagle's medium (DMEM) was obtained from Pan Biotech (Aidenbach, Germany). Fetal bovine serum (FBS), L‐glutamine, and TrypLE reagent were bought from Gibco (Thermo Fisher Scientific, Paisley, UK). Phosphate buffered saline (PBS) was purchased from Lonza (Basel, Switzerland). Zeocin was supplied by Thermo Fisher Scientific (Waltham, MA, USA) and blasticidin from PAA Laboratories GmbH (GE Healthcare, Chicago, IL, USA).

Water was procured from a Millipore Milli‐Q^TM^ reference ultrapure water purification system (Merck KGaA). KHB was prepared monthly with the addition of 25 mM sodium hydrogencarbonate, 25 mM HEPES, and 2 mM calcium chloride dihydrate (pH 7.4) and preserved at 4 °C.

The synthesis of the substrates gstachamine and gstachidine, along with their corresponding nonvolatile metabolites 2‐(3‐(sec‐butylamino)‐1‐phenylpropyl)‐4‐methylphenol (*N*‐dealkylated gstachamine) and 2‐(3‐(butylamino)‐1‐phenylpropyl)‐4‐methylphenol (*N*‐dealkylated gstachidine), has been outlined in the *supplementary information*.

##### HepG2 Cell Clones

HepG2 cell clones overexpressing CYP3A4, CYP2C9, or CYP2D6 along with their respective empty vector (EV) control cell lines were supplied by the Molecular Cell Biology Group at the Institute of Biotechnology, Brandenburg University of Technology Cottbus‐Senftenberg, Senftenberg, Germany.^[^ ^19^, ^20^, ^21^ ^]^ HepG2 cells were cultivated in DMEM (completed with 10% FBS as well as 2 mM L‐glutamine) in a CB 170 humidified incubator (Binder, Tuttlingen, Germany) at 37 °C with 5% CO_2_. Cells were passaged when they reached approximately 85% confluency, at 5‐ to 7‐day intervals.

Following thawing, the cells were selected at three‐ to four‐week intervals by the addition of blasticidin to the media (3 µg mL^−1^) of the CYP3A4‐overexpressing and EV cells and zeocin to the DMEM (300 µg mL^−1^) of the CYP2D6 and CYP2C9 overexpressing and EV cells. In all instances throughout the course of the experiments, no more than 1% DMSO, used as solvent for different compounds, was added to the cells. Cells were tested for mycoplasma infection on a regular basis.

##### Biotransformation of Gstachamine, Gstachidine, and Tolterodine with HepG2 Cell Clones

Cells (5 × 10^5^ per well) were seeded into 24‐well plates 24 h before start of the experiment. For volatile metabolite analysis, cells (2 × 10^6^ per flask) were seeded into T75 flasks (250 mL, Greiner Bio‐One, Kremsmünster, Austria) and further cultured for 5 days to reach approximately 95% confluence.

After incubation, medium was removed and cells were washed with KHB. Then, cells were treated with 110 µM gstachamine, gstachidine, or tolterodine in KHB at 37 °C and 5% CO_2_. The concentration dependent effects of gstachamine were performed according to Stock et al.,^[^ ^9^ ^]^ with following modifications: cells were treated with different concentrations (0 µM, 55 µM, 110 µM, 150 µM, 200 µM, 250 µM, 300 µM, and 350 µM) of gstachamine diluted in KHB. The inhibitory effect was investigated using the pan‐CYP inhibitor 1‐ABT and the CYP3A4 inhibitor ketoconazole. The cells were treated with 110 µM gstachamine in KHB, which had been supplemented with either 1000 µM 1‐ABT or 5 µM ketoconazole. Ketoconazole was preincubated separately for 10 min at 37 °C prior to the addition of 110 µM gstachamine in KHB to the cells.

The incubation time varied depending on the experiment. In order to analyze the comparison between gstachamine and gstachidine, cells were incubated for a period of 260 min. For investigating the time dependence of *N*‐dealkylated gstachamine production, incubation time ranged from 30 to 260 min, while for investigating the biotransformation in different HepG2 cell clones, concentration‐dependent effects, and CYP inhibitors, the incubation time was set to 150 min.

For the headspace analysis, T75 cell culture flasks were sealed airtight using the sampling device outlined by Stock et al. .^[^ ^9^ ^]^ Following incubation, 500 µL of the liquid and 150 mL of the gaseous phases above the cells were collected and prepared for analysis using liquid chromatography mass spectrometry (LC‐MS) and proton transfer reaction‐time of flight‐mass spectrometry (PTR‐ToF‐MS), respectively (for further details, see *sample preparation* section).

Furthermore, the nonenzymatic degradation of the substrate (w/o cells) was measured in either T75 cell culture flasks or 24‐well plates, which were treated in the same way as the cells, except that no cells were cultured in them.

##### Equilibrium between Liquid and Gas Phase

To investigate the correlation between butanone levels in the buffer solution and in the headspace, a butanone solution was prepared to match the amount of *N*‐dealkylated gstachamine produced after 150 min of biotransformation. For this purpose, 2 µM of butanone in KHB were transferred to a T75 cell culture flask and sealed airtight using the sampling device. The flask was incubated in the cell incubator for 150 min, and the sample was subsequently prepared as described in *sample preparation* section and measured using PTR‐ToF‐MS.

##### Preparation of Calibration Standards

External calibrations of the nonvolatile metabolites of gstachamine, gstachidine, and tolterodine were carried out by dissolving each metabolite in DMSO yielding 10 mM stock solutions. Subsequently, the stock solutions were diluted with 1:1 ACN/H_2_O (v/v) to simulate yield the concentration of the calibration points. Each concentration point (0.05 µM, 0.1 µM, 0.5 µM, 1 µM, 2 µM, and 3 µM) was then measured by LC‐MS.

The total evaporation method was used to calibrate the volatile metabolite butanone as outlined by Stock et al.^[^ ^9^ ^]^ In this case, 0.5 µL butanone was injected into the preheated gas bulb. The resulting nine calibration standards (0 µM, 0.5 µM, 0.7 µM, 1.0 µM, 1.4 µM, 2.1 µM, 2.8 µM, 3.5 µM, and 4.2 µM) were then measured by PTR‐ToF‐MS.

##### Sample Preparation

After given incubation times, cell supernatants were collected, mixed 1:1 with cold ACN, and then centrifuged using an Eppendorf 5415 R microcentrifuge (Eppendorf, Hamburg, Germany) for 5 min at 16,100 relative centrifugal force. Afterwards, the sample was filtered through a PTFE syringe filter with a pore size of 0.2 µm (Agilent Technologies, Santa Clara, CA, USA) to ensure the removal of any remaining solids. The samples were then analyzed by LC‐MS.

For the analysis of the headspace above the cells after given incubation times, 150 mL of the gaseous phase from the cell culture flask was drawn into a glass syringe (250 mL, Socorex Isba SA, Ecublens, Switzerland) by exchanging the gas volume in the flask with nitrogen (purity at least 99.7%, NGM 11 nitrogen generator, cmc Instruments GmbH, Eschborn, Germany). Two glass syringes were therefore connected to the sampling device: one syringe contained more than 150 mL of nitrogen, while the other collected the gaseous phase as nitrogen was introduced into the flask. The samples were subsequently analyzed using PTR‐ToF‐MS.

##### LC‐MS Analysis

For LC‐MS analysis, a Vanquish UHPLC Flex system (Thermo Fisher Scientific) with a binary pump coupled to an Orbitrap Q‐Exactive system was used. Separation was carried out at 40 °C using a ZORBAX Eclipse XDB‐C18 column (1.8 μm, 2.1 × 100 mm) guarded by a ZORBAX Eclipse Plus C18 guard column (1.8 μm, 2.1 × 5 mm), both from Agilent Technologies. The flow rate was adjusted to 0.3 mL min^−1^ using water (eluent A) and acetonitrile (eluent B), each supplemented with 0.1% formic acid as the mobile phase. The subsequent gradient, described in Stock et al.,^[^ ^10^ ^]^ was performed: 0 min/20% B, 0.8 min/20% B, 5.8 min/90% B, 7.5 min/90% B, 7.9 min/20% B, 11.6 min/20% B. The injected sample volume was 1 µL and the temperature of the sample tray was 6 °C.

Mass spectrometric measurements were carried out in positive ionization mode with a heated electro spray ionization (HESI) source. Nitrogen was used as an auxiliary, sheath, and sweep gas, and the spray voltage was set to 3.5 kV. The capillary and auxiliary gas temperature were set to 300 °C and 350 °C, respectively. Data acquisition was carried out using a targeted single ion monitoring (t‐SIM) and a full mass scan. The mass resolution (*m/Δm*) was 70,000 for the t‐SIM. For the full‐mass scan, the resolution was 35,000 with a scan range of *m/z* 100–500. Quantification of the nonvolatile metabolites was performed using the protonated single ion at 298.217 *m/z* for *N*‐dealkylated gstachamine and gstachidine (C_20_H_28_NO^+^) and 284.201 *m/z* for *N*‐dealkylated tolterodine (C_19_H_26_NO^+^). The software QuanBrowser (Thermo Xcalibur 4.2.47, Thermo Fisher Scientific) was used for data processing.

##### PTR‐ToF‐MS Analysis

Analysis of the volatile metabolites were performed on a PTR‐ToF 6000 X2 (Ionicon Analytik GmbH, Innsbruck, Austria). The PTR‐ToF‐MS was operated with a drift voltage of 417 V, a drift tube pressure of 2.6 mbar, and a drift tube temperature of 100 °C, resulting in a reduced electric field strength *E/N* of 120 Townsend. A hollow cathode was used with a current of 4 mA, generating H_3_O^+^ reagent ions. The measured sample inlet flow was adjusted to approximately 53 mL min^−1^. Data acquisition consisted of recording a spectrum at 1000 ms intervals, with measurements performed for a minimum duration of 70 s.

The PTR‐MS Viewer software (version 3.4.4.22, Ionicon Analytik GmbH) was used for data processing and evaluation. Mass spectral peaks were first fitted using a pseudo‐Voigt profile, and then a two‐point mass calibration was performed using a signal of the primary reagent ion at *m/z* 21.023 (H_3_^18^O^+^) and an internal diiodobenzene standard signal at *m/z* 203.943 (C_6_H_5_I^+^). To account for variations in the reagent ion signal between different measurements, the product ion intensities of butanone (C_4_H_9_O^+^) at *m/z* 73.065 were normalized to a reagent ion intensity of 10^6^ ions per second (ncps), using the H_3_^18^O^+^ signal at *m/z* 21.023 and the water cluster H_2_O.H_3_^18^O^+^ signal at *m/z* 39.033. The average signal intensities of the ncps were determined based on 30 sample points at the end of the measurement, during which a stable ion signal was reached.

##### Statistical Analyses

All cell‐based data herein show the mean ± one standard deviation of a minimum of two independent experiments in 24‐well plates or cell culture flasks, each comprising separate duplicate or triplicate measurements. One‐way ANOVA with Tukey's multiple comparison test was used to test for significant differences between groups using OriginPro (version 2022b, OriginLab, Northampton, MA, USA). Statistical significance was assumed for *p* < 0.05 for all statistical analyses. The determination of the TC_50_ (half maximal toxic concentration) values was achieved using Prism 8.0 (GraphPad Software Inc., San Diego, CA, USA).

Limits of detection (LOD) and quantification (LOQ) of *N*‐dealkylated gstachamine and butanone were calculated based on a calibration with equidistant increments ranging from 0 µM to 0.02 µM for LC‐MS and 0 nM to 1.4 nM for PTR‐ToF‐MS analyses. The procedure followed the German industry standard DIN32645: 2008–11 guideline.^[^ ^22^ ^]^

## Supporting Information

The authors have cited additional references within the Supporting Information.^[^ ^23^ ^]^

## Conflict of Interest

The authors declares no conflicts of interest.

## Supporting information

Supplementary Material

## Data Availability

The data that support the findings of this study are available from the corresponding author upon reasonable request.
