# Supplementary material for: Towards the Use of Metabolic Volatiles in Breath for Determining Drug Response: Gstachamine as an Unlabeled Substrate to Measure CYP3A4 Activity
Source: ChemMedChem. 2025 Aug 25;20(18):e202500492. doi: 10.1002/cmdc.202500492 (PMC12479387; doi:10.1002/cmdc.202500492)
Supplement: Supplementary file 1 — Supplementary Material [file CMDC-20-e202500492-s001.pdf]

Supplementary Information for

Towards the Use of Metabolic Volatiles in Breath for Determining Drug Response: Gstachamine as an Unlabeled Substrate to Measure CYP3A4 Activity

Valentina Stock[1], Rebecca Hofer[1], Klaus R. Liedl[2], Jakob Troppmair[3], Thierry Langer[4], Hubert Gstach[4], Christian Dank[4,5], Sarah Kammerer[1,6], Veronika Ruzsanyi[1]*

1 Institute for Breath Research, University of Innsbruck, Innrain 80/82, 6020 Innsbruck, Austria

2 Department of General, Inorganic and Theoretical Chemistry, University of Innsbruck, Innrain 80/82, 6020 Innsbruck, Austria

3 Daniel Swarovski Research Laboratory, Department of Visceral, Transplant and Thoracic Surgery, Medical University of Innsbruck, Innrain 66, 6020 Innsbruck, Austria

4 Department of Pharmaceutical Chemistry, University of Vienna, Althanstraße 14, 1090 Vienna, Austria

5 Institute of Organic Chemistry, University of Vienna, Währinger Straße 38, 1090 Vienna, Austria

6 Institute of Biotechnology, Molecular Cell Biology, Brandenburg University of Technology Cottbus-Senftenberg, 01968 Senftenberg, Germany

*Corresponding author: Veronika.Ruzsanyi@uibk.ac.at

Experimental

Reaction monitoring and purification of compounds

Thin layer chromatography (TLC)

Reaction monitoring was performed by thin layer chromatography (TLC) on Merck silica gel 60-F_{254} glass plates or on Macherey & Nagel POLYGRAM SIL G/UV 254 aluminum foils. The plates were developed with mixtures of hexane/ethyl acetate and methanol/ethyl acetate. Compound spots were visualized by UV (254 nm) irradiation or by staining in a container equipped with iodine adsorbed on silica gel.

Middle pressure liquid chromatography (MPLC)

Purifications of compounds were performed by middle pressure liquid chromatography (MPLC) on silica gel 60 from Merck (0.040-0.063 µm, 240-400 mesh). The unique home built MPLC system was provided by Hubert Gstach and consisted of columns,

an FMI pump (Fluid Metering, Inc., Syosset, Nassau County, NY, United States) and an Amersham Superfrac fraction collector.

Analytical characterization

High resolution mass spectroscopy (HRMS)

Mass spectra were obtained using a 7200B GC/Q-TOF spectrometer from Agilent, using electron ionization (EI), and an Orbitrap Exploris 120 (Thermo Scientific), using electrospray ionization (ESI).

Chemicals and building blocks

Solvents were purchased from Sigma-Aldrich and used without further purification. Ethyl acetate and *n*-hexane were distilled prior use in chromatographic separations.

Amine components and 3-(2-Methoxy-5-methylphenyl)-3-phenylpropyl 4-methylbenzenesulfonate were used as mixtures of stereoisomers. The resulting compounds are thus also mixtures of stereoisomers.

Nuclear magnetic resonance spectroscopy (NMR)

NMR spectra were recorded at the NMR centre of the Faculty of Chemistry, University of Vienna, on an Avance III HDX 700 spectrometer equipped with a quadruple QCIF cryoprobe (700.40 MHz for ^{1}H, 176.12 MHz for ^{13}C). The software used for processing of 1D- (^{1}H, ^{13}C) and 2D- (COSY, HMBC, HSQC) NMR spectra was MestReNova. Coupling constants (J) are given in Hertz (Hz) and refer to the first order interpretation (apparent coupling constants J_{app} are provided). Assignment of resonances was performed with COSY, HSQC and HMBC respectively. ^{x}J refers to homonuclear HH-coupling over x bonds. $^{x}J_{CF}$ refers to heteronuclear CF-coupling over x bonds. Solvents used for NMR spectroscopy: DMSO-d_6, hexadeuterio dimethyl sulfoxide (CAS RN 2206-27-1). Chemical shift calibration [23]: DMSO-d_6, ^{1}H δ = 2.50, ^{13}C δ = 39.52 ppm. NMR characterization: br, broad; multiplicity: m, multiplet; s, singlet; d, doublet; t, triplet; q, quaternary or quartet; qu, quintet; hept, heptet; AA`BB`: the four most intense peaks are given; AA´ or BB´ part of AA´BB´: m[e.g. t, dd, q, tt, sext] notation in brackets describes the overall appearance of the signal pattern;
2D NMR techniques used for the assignment of ^{1}H and ^{13}C resonance signals: HSQC, Heteronuclear Single Quantum Coherence; HMBC, Heteronuclear Multiple Bond Correlation; COSY, Correlation Spectroscopy.

Synthetic procedures

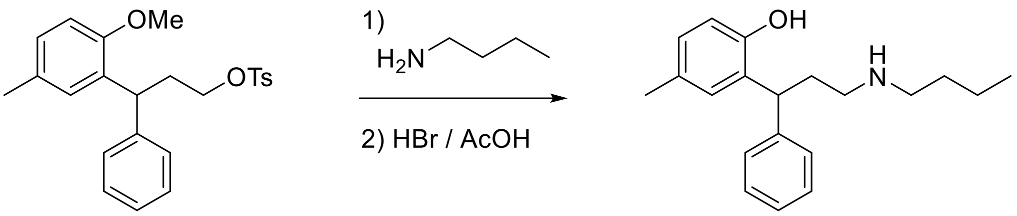

***N*-dealkylated Gstachidine**

To a solution of 3-(2-methoxy-5-methylphenyl)-3-phenylpropyl 4-methylbenzenesulfonate (4.218 g, 10.27 mmol, 1 eq.) in 30 mL of ACN, was added *n*-butyl amine (1,503 g, 20.5 mmol, 2 eq.). The reaction mixture was heated to reflux for 48 hours. The reaction mixture was concentrated under reduced pressure and the resulting yellow to orange oil. Column chromatography (silica, hexanes: ethyl acetate 4:1) gave the desired secondary amine (1.89 g, 6.06 mmol, 59% yield).

N-(3-(2-Methoxy-5-methylphenyl)-3-phenylpropyl)butan-1-amine (790 mg, 2.54 mmol, 1 eq) was suspended in 5 mL of acetic acid, then 3 mL of HBr (48% in water) were added. The reaction mixture was stirred at 100°C for 18h. The reaction mixture was concentrated under reduced pressure and the resulting grey crystalline material was washed with 60 mL of ethyl acetate to give 700 mg colourless crystals (93 % yield) of *N*-dealkylated Gstachidine as hydrobromide salt.

HRMS (EI) from hydrobromide for $C_{20}H_{28}NO$ [M^+] calculated: 298.2171, found: 298.2161.

^{1}H NMR (600 MHz, DMSO-d$_6$, 25 °C) δ = 9.16 (br s, 1H), 8.52 (br s, 2H), 7.28 (d, *J* = 4.4 Hz, 4H), 7.20 – 7.14 (m, 1H), 6.96 (d, *J* = 2.2 Hz, 1H), 6.81 (dd, *J* = 8.2, 2.2 Hz, 1H), 6.70 (d, *J* = 8.1 Hz, 1H), 4.31 (t, *J* = 7.9 Hz, 1H), 2.90 – 2.83 (m, 2H), 2.77 (qt, *J* = 12.2, 7.7 Hz, 2H), 2.33 (q, *J* = 8.0 Hz, 2H), 2.17 (s, 3H), 1.57 – 1.48 (m, 2H), 1.30 (h, *J* = 7.4 Hz, 2H), 0.87 (t, *J* = 7.4 Hz, 3H).

^{13}C{^{1}H}NMR (151 MHz, DMSO-d$_6$, 25°C) δ = 152.25, 143.82, 129.33, 128.30, 127.87, 127.72, 127.58, 127.47, 126.08, 115.14, 46.33, 45.84, 40.47, 30.20, 27.56, 20.41, 19.24, 13.48.

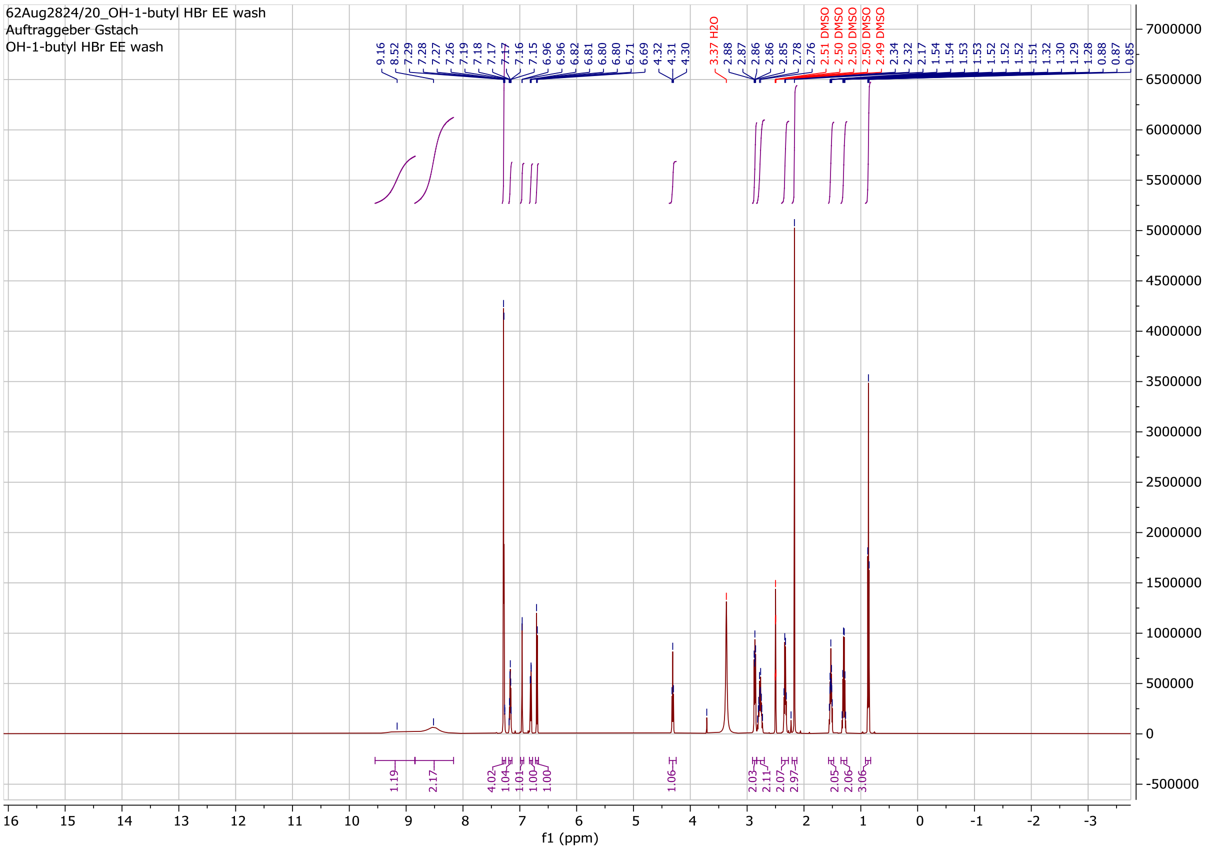
62Aug2824/20_OH-1-butyl HBr EE wash
Auftraggeber Gstach
OH-1-butyl HBr EE wash
3.37 H2O
2.51 DMSO
2.50 DMSO
2.50 DMSO
2.50 DMSO
2.49 DMSO
f1 (ppm)

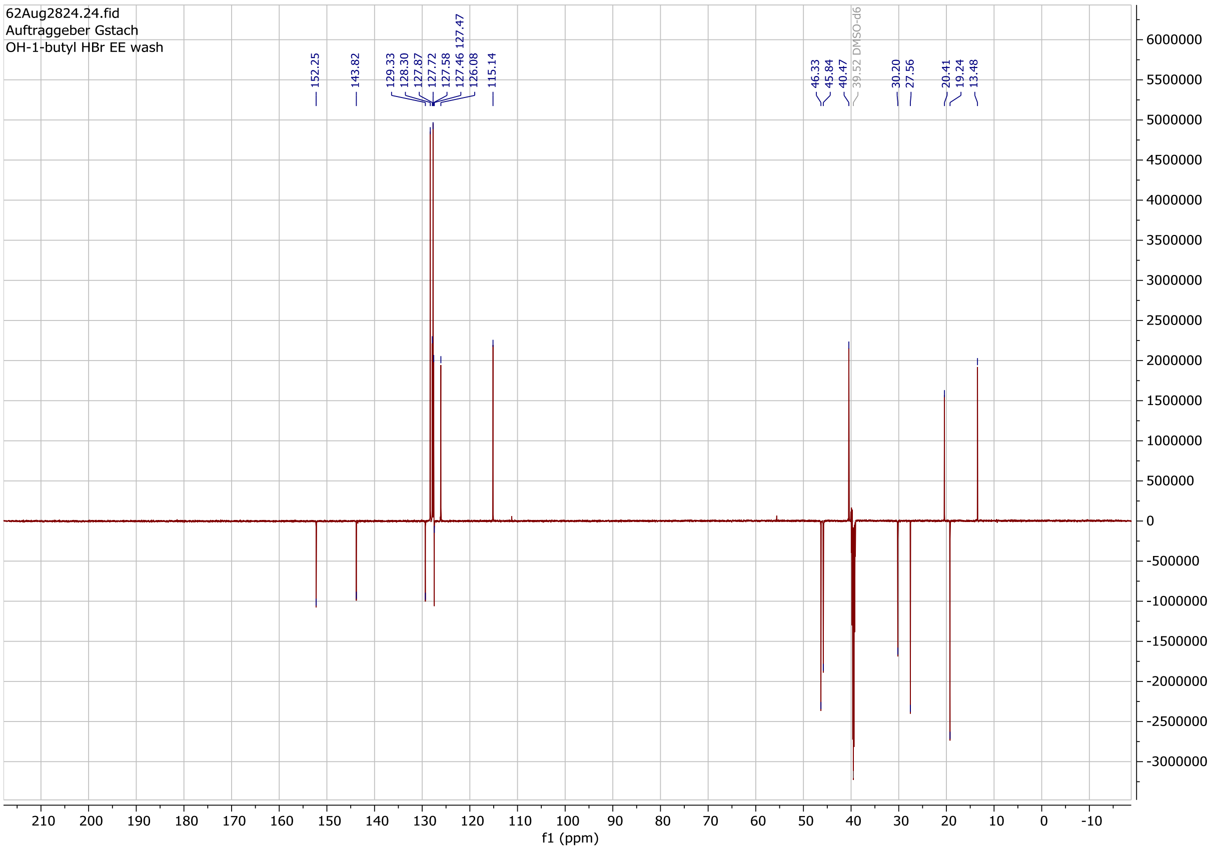
62Aug2824.24.fid
Auftraggeber Gstach
OH-1-butyl HBr EE wash
152.25
143.82
129.33
128.30
127.87
127.72
127.58
127.47
127.46
126.08
115.14
46.33
45.84
40.47
39.52 DMSO-d6
30.20
27.56
20.41
19.24
13.48
f1 (ppm)

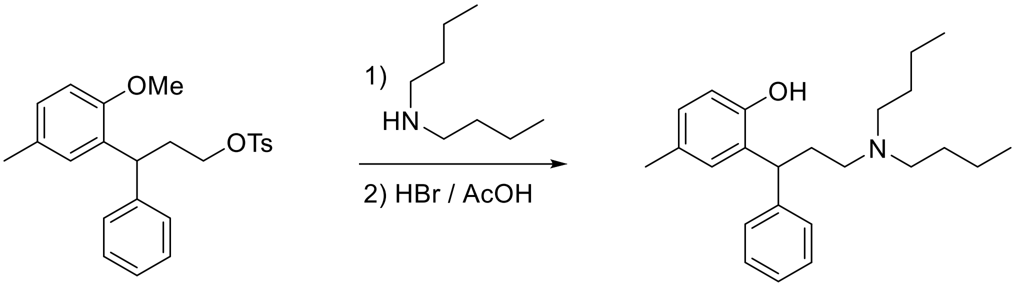

Gstachidine

To a solution of 3-(2-methoxy-5-methylphenyl)-3-phenylpropyl 4-methylbenzenesulfonate (2.25 g, 5.48 mmol, 1 eq.) in 16 mL of ACN, was added di-*n*-butyl amine (1,47 g, 11.0 mmol, 2 eq.). The reaction mixture was heated to reflux for 44 hours. The reaction mixture was concentrated under reduced pressure and the resulting slightly coloured semicrystals (3.28 g) were completely dissolved in ethyl acetate. Trituration with n-hexane gave solids which were filtered off, washed with ethyl acetate and twice with n-hexane. The filtrate was then concentrated under reduced pressure yielding 1.48g of crude product which was treated with 19.5 mL of acetic acid and 3.3 mL of HBr (48% in water). The reaction mixture was stirred at 100°C for 18h. The reaction mixture was concentrated under reduced pressure and the resulting crystalline material was washed with ~50 mL of ethyl acetate to give 898 mg colourless crystals of *N*-dealkylated Gstachidine as hydrobromide salt.

The free base was obtained from the pure hydrobromide by turbinating the water insoluble hydrobromide in a mixture of ethyl acetate and saturated sodium bicarbonate at room temperature until all solid material was dissolved. The organic phase was separated, washed neutral with water and brine, dried over sodium sulfate. The clear ethyl acetate phase was concentrated on a rotary evaporator to give Gstachamine as free base (711 mg, 37 % yield over 3 steps)

^{1}H NMR (600 MHz, DMSO-d$_6$, 25 °C) δ = 7.28 – 7.20 (m, 5H), 7.13 – 7.09 (m, 1H), 6.96 (d, J = 2.2 Hz, 1H), 6.77 – 6.73 (m, 1H), 6.64 (d, J = 8.1 Hz, 1H), 4.29 (t, J = 7.8 Hz, 1H), 2.43 – 2.37 (m, 4H), 2.37 – 2.31 (m, 2H), 2.12 – 2.01 (m, 2H), 1.34 – 1.15 (m, 9H), 0.82 (t, J = 7.1 Hz, 6H).

^{13}C{^{1}H}NMR (151 MHz, DMSO-d$_6$, 25°C) δ = 152.48, 145.11, 130.66, 128.13, 128.00, 127.96, 127.31, 127.15, 125.73, 115.14, 53.03, 51.45, 40.57, 30.97, 28.39, 20.47, 20.07, 13.97.

HRMS (EI) from hydrobromide for $C_{24}H_{36}NO$ [M$^+$] calculated: 354.2797, found: 354.2782.

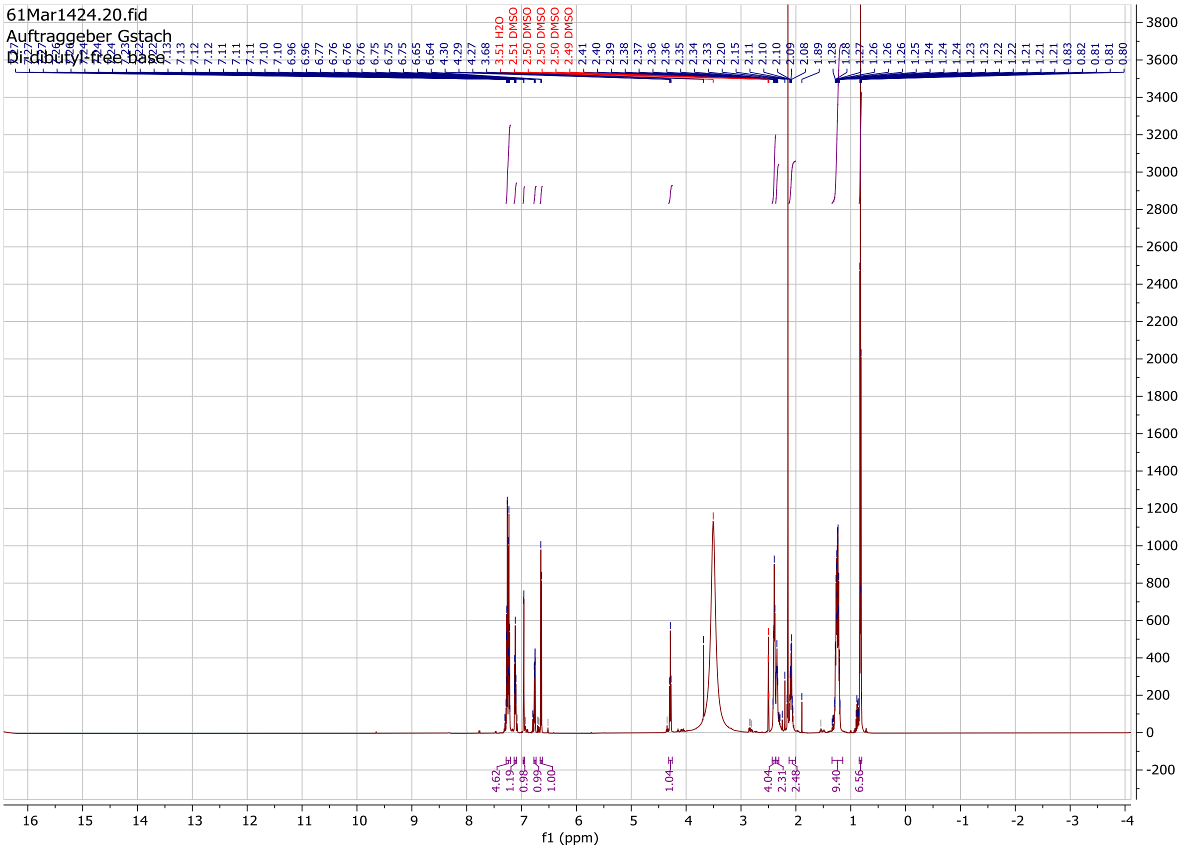
61Mar1424.20.fid
Auftraggeber Gstach
Di-dibutyl-free base
f1 (ppm)

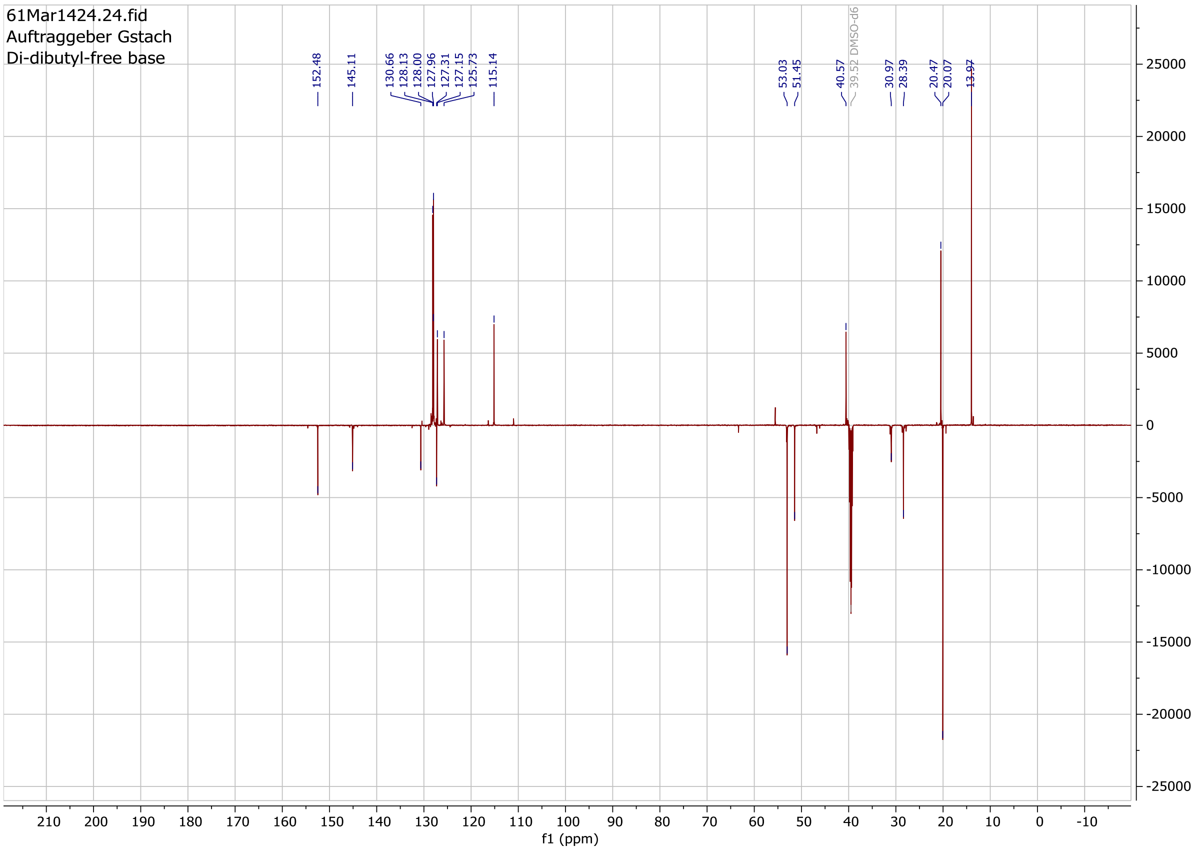
61Mar1424.24.fid
Auftraggeber Gstach
Di-dibutyl-free base
152.48
145.11
130.66
128.13
128.00
127.96
127.31
127.15
125.73
115.14
53.03
51.45
40.57
39.52 DMSO-d6
30.97
28.39
20.47
20.07
13.97
f1 (ppm)

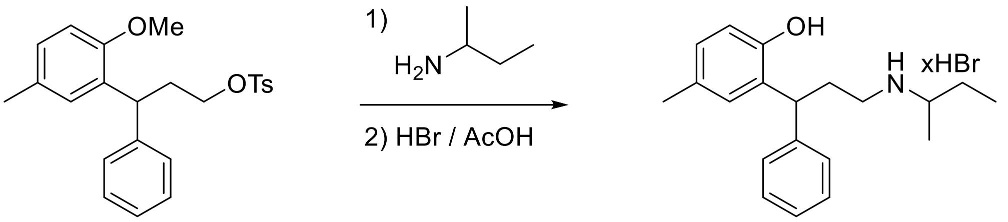

***N*-dealkylated Gstachamine**

To a solution of 3-(2-methoxy-5-methylphenyl)-3-phenylpropyl 4-methylbenzenesulfonate (2.12 g, 5.16 mmol, 1 eq.) in 15 mL of ACN, was added sec-butyl amine (755 mg, 10.3 mmol, 2 eq.). The reaction mixture was heated to reflux for 52 hours. The reaction mixture was concentrated under reduced pressure and the resulting oil was partitioned between 10 mL of 2M NaOH and 20 mL of DCM and the layers were separated. The organic layer was washed three times with water, followed by brine, and dried over Na_2SO_4. The resulting liquid (1.764 g) was suspended in 10 mL of acetic acid and 6 mL of HBr (48% in water) were added. The reaction mixture was stirred at 100°C for 18h. The reaction mixture was concentrated under reduced pressure and the resulting grey crystalline material was washed with 60 mL of ethyl acetate to give 809 mg colourless crystals (50 % yield) of *N*-dealkylated Gstachamine as hydrobromide salt.

HRMS (EI) from hydrobromide for $C_{20}H_{28}NO$ [M^+] calculated: 298.2171, found: 298.2159.

^{1}H NMR (600 MHz, DMSO-d$_6$, 25 °C) δ = 9.23 (br s, 1H), 8.48 (br s, 2H), 7.33 – 7.24 (m, 4H), 7.21 – 7.13 (m, 1H), 6.98 (d, *J* = 2.3 Hz, 1H), 6.81 (dd, *J* = 8.0, 2.2 Hz, 1H), 6.70 (d, *J* = 8.1 Hz, 1H), 4.34 (t, *J* = 7.9 Hz, 1H), 3.17 – 3.06 (m, 1H), 2.89 – 2.69 (m, 2H), 2.43 – 2.27 (m, 2H), 2.17 (s, 3H), 1.77 – 1.64 (m, 1H), 1.49 – 1.35 (m, 1H), 1.14 (dd, *J* = 6.5, 2.1 Hz, 3H), 0.93 – 0.78 (m, 3H).

^{13}C{^{1}H}NMR (151 MHz, DMSO-d$_6$, 25°C) δ =152.27, 152.24, 143.87, 143.81, 129.37, 129.28, 128.33, 128.26, 127.86, 127.84, 127.72, 127.69, 127.55, 127.43, 126.04, 115.14, 115.12, 54.13, 42.87, 40.51, 40.46, 30.30, 25.28, 25.21, 20.38, 15.03, 14.99, 9.46, 9.44.

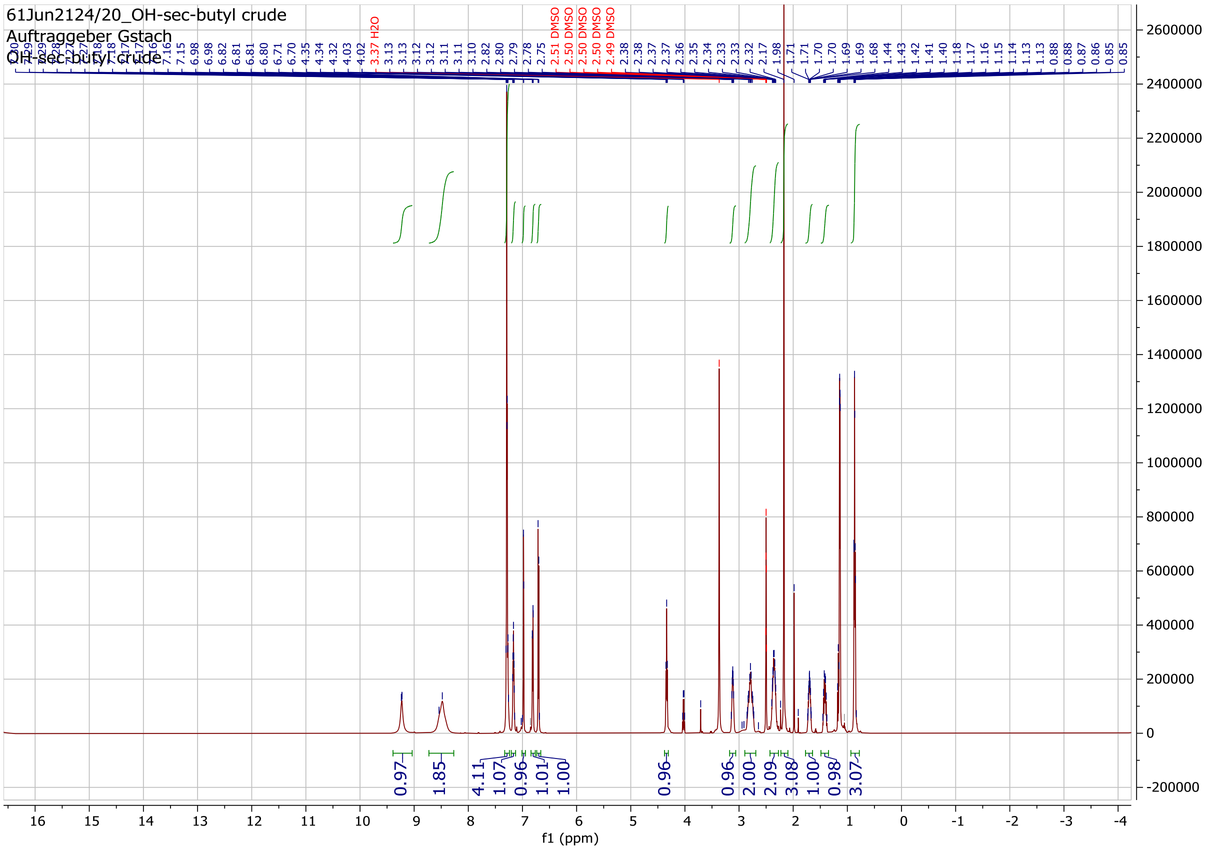
61Jun2124/20_OH-sec-butyl crude
Auftraggeber Gstach
OH-sec-butyl crude
7.15
6.98
6.98
6.82
6.81
6.81
6.80
6.71
6.70
4.35
4.34
4.32
4.03
4.02
3.37 H2O
3.13
3.13
3.12
3.12
3.11
3.11
3.10
2.82
2.80
2.79
2.78
2.75
2.51 DMSO
2.50 DMSO
2.50 DMSO
2.50 DMSO
2.49 DMSO
2.38
2.38
2.37
2.37
2.36
2.35
2.34
2.33
2.33
2.32
2.17
1.98
1.71
1.71
1.70
1.69
1.69
1.68
1.44
1.43
1.42
1.41
1.40
1.18
1.17
1.16
1.15
1.14
1.13
1.13
0.88
0.87
0.86
0.85
0.85
0.97
1.85
4.11
1.07
0.96
1.01
1.00
0.96
0.96
2.00
2.09
3.08
1.00
0.98
3.07
f1 (ppm)

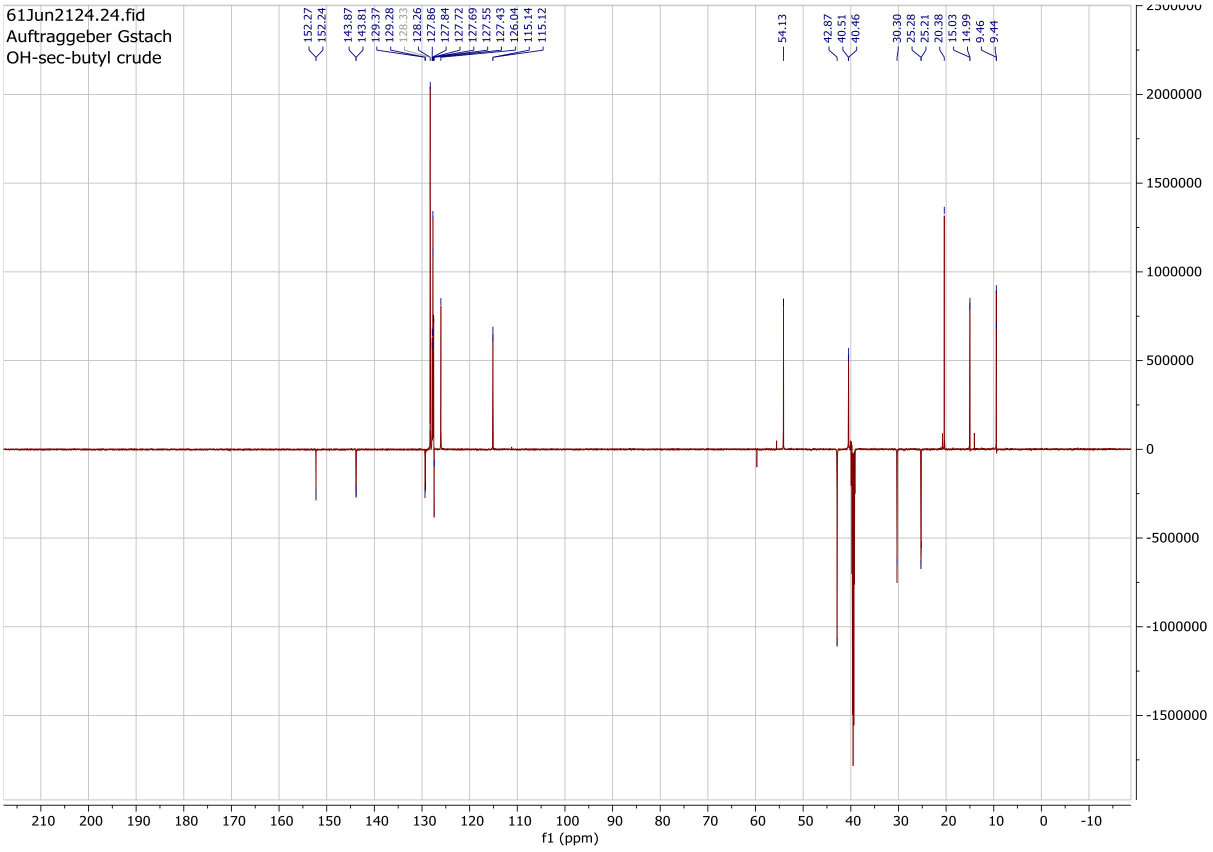
61Jun2124.24.fid
Auftraggeber Gstach
OH-sec-butyl crude
152.27
152.24
143.87
143.81
129.37
129.28
128.33
128.26
127.86
127.84
127.72
127.69
127.55
127.43
126.04
115.14
115.12
54.13
42.87
40.51
40.46
30.30
25.28
25.21
20.38
15.03
14.99
9.46
9.44
f1 (ppm)

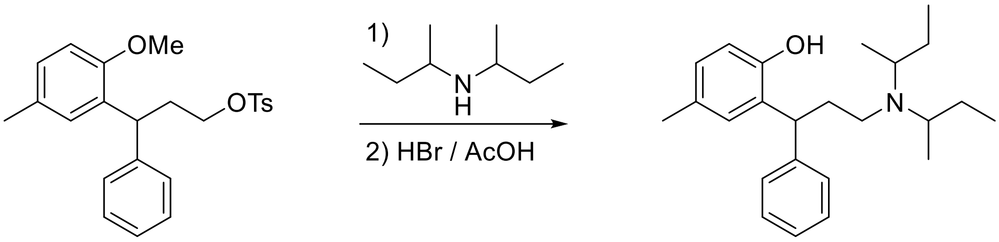

Gstachamine

To a solution of 3-(2-Methoxy-5-methylphenyl)-3-phenylpropyl 4-methylbenzenesulfonate (4.5 g, 11.0 mmol, 1 eq.) in 32 mL of ACN, was added di-sec-butyl amine (2.83 g, 21.9 mmol, 2 eq.). The reaction mixture was heated to reflux for 55 hours. The reaction mixture was concentrated under reduced pressure and the resulting oil was partitioned between 10 mL of 2M NaOH and 20 mL of DCM and the layers were separated. The organic layer was washed three times with water, followed by brine, and dried over Na2SO4. After removal of volatile components under reduced pressure, the resulting residue (~8 g) was suspended in 20 mL of acetic acid and 10 mL of HBr (48% in water) were added. The reaction mixture was stirred at 100°C for 18h. The reaction mixture was concentrated under reduced pressure and the resulting crystalline material was treated with 2 M NaOH and DCM, whereas the pH was set to 11. The organic layers were washed with water until neutral and the organic layer was then dried over Na_2SO_4 and concentrated under reduced pressure. The free base was then purified by column chromatography (silica, hexane : ethyl acetate gradient). The fractions containing mainly product were pooled and concentrated under reduced pressure to give a residue (~530 mg). The residue was washed with n-hexane and the remaining solid (30 mg, 1% yield) was identified as the free base of Gstachamine.

^{1}H NMR (600 MHz, DMSO-d$_6$, 25 °C) δ = 9.03 (br s, 1H), 7.29 – 7.18 (m, 4H), 7.14 – 7.07 (m, 1H), 6.99 – 6.91 (m, 1H), 6.78 – 6.72 (m, 1H), 6.63 (d, *J* = 8.0 Hz, 1H), 4.38 – 4.24 (m, 1H), 2.64 – 2.53 (m, 2H), 2.38 – 2.25 (m, 2H), 2.18 – 2.13 (m, 3H), 2.13 – 1.91 (m, 2H), 1.38 – 1.20 (m, 2H), 1.16 – 1.02 (m, 2H), 0.88 – 0.77 (m, 12H).

^{13}C{^{1}H}NMR (151 MHz, DMSO-d$_6$, 25°C) δ = 152.44, 152.31, 152.23, 145.63, 145.36, 145.17, 131.14, 130.85, 130.52, 127.96, 127.89, 127.84, 127.82, 127.80, 127.79, 127.76, 127.03, 127.01, 126.88, 126.84, 126.81, 125.46, 125.44, 114.87, 114.86, 114.84, 54.43, 54.28, 53.66, 53.50, 43.00, 42.68, 42.39, 40.65, 40.43, 40.23, 35.33, 35.01, 34.78, 28.55, 28.49, 27.51, 27.33, 21.08, 20.41, 20.38, 17.93, 17.88, 17.85, 16.96, 16.89, 11.86, 11.83.

HRMS (EI) from hydrobromide for $C_{24}H_{36}NO$ [M^+] calculated: 354.2797, found: 354.2784.

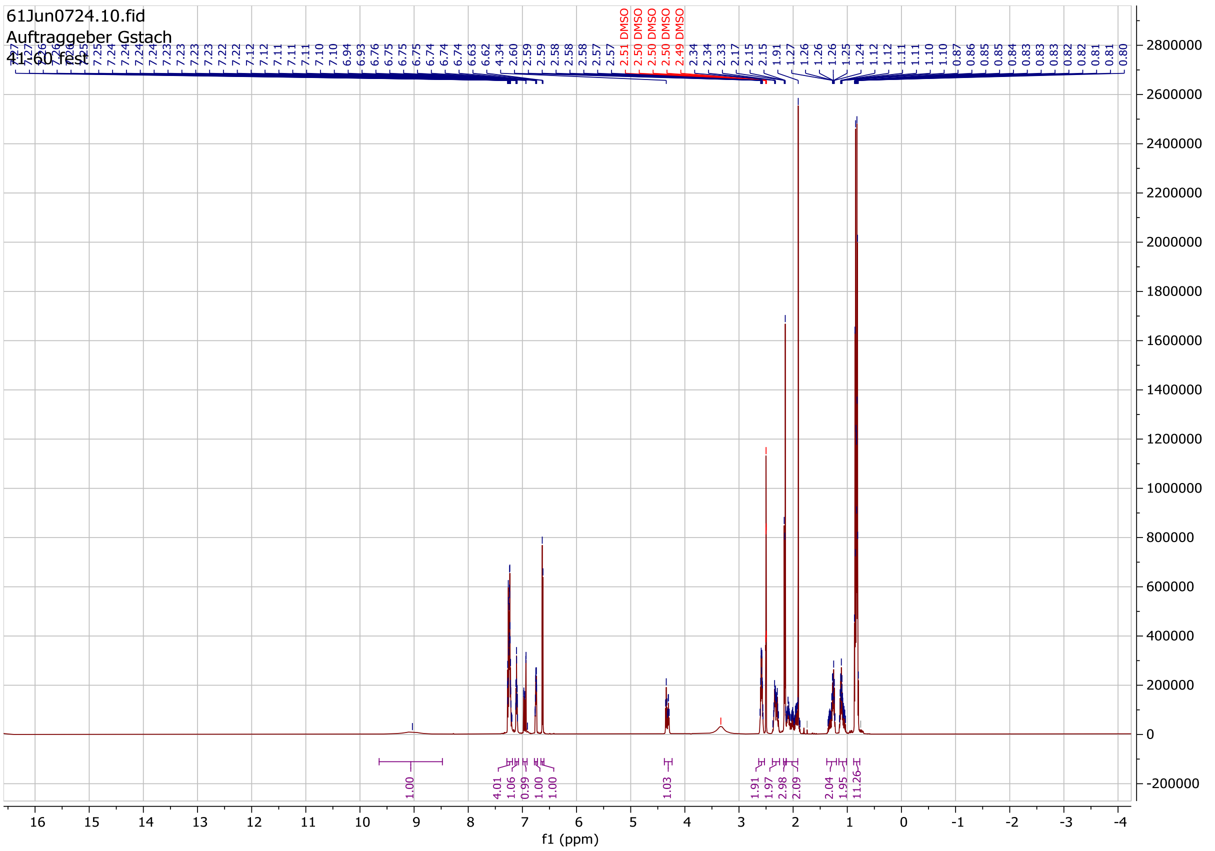
61Jun0724.10.fid
Auftraggeber Gstach
41-60 fest
7.25 7.24 7.24 7.24 7.24 7.23 7.23 7.23 7.23 7.22 7.22 7.12 7.12 7.11 7.11 7.11 7.10 7.10 6.94 6.93 6.76 6.75 6.75 6.75 6.74 6.74 6.74 6.63 6.62 4.34 2.60 2.59 2.59 2.58 2.58 2.58 2.57 2.57
2.51 DMSO 2.50 DMSO 2.50 DMSO 2.50 DMSO 2.49 DMSO
2.34 2.34 2.33 2.17 2.15 2.15 1.91 1.27 1.26 1.26 1.26 1.25 1.24 1.12 1.12 1.11 1.11 1.10 0.87 0.86 0.85 0.85 0.84 0.83 0.83 0.83 0.82 0.82 0.81 0.81 0.80
1.00 4.01 1.06 0.99 1.00 1.00 1.03 1.91 1.97 2.98 2.09 2.04 1.95 11.26
f1 (ppm)
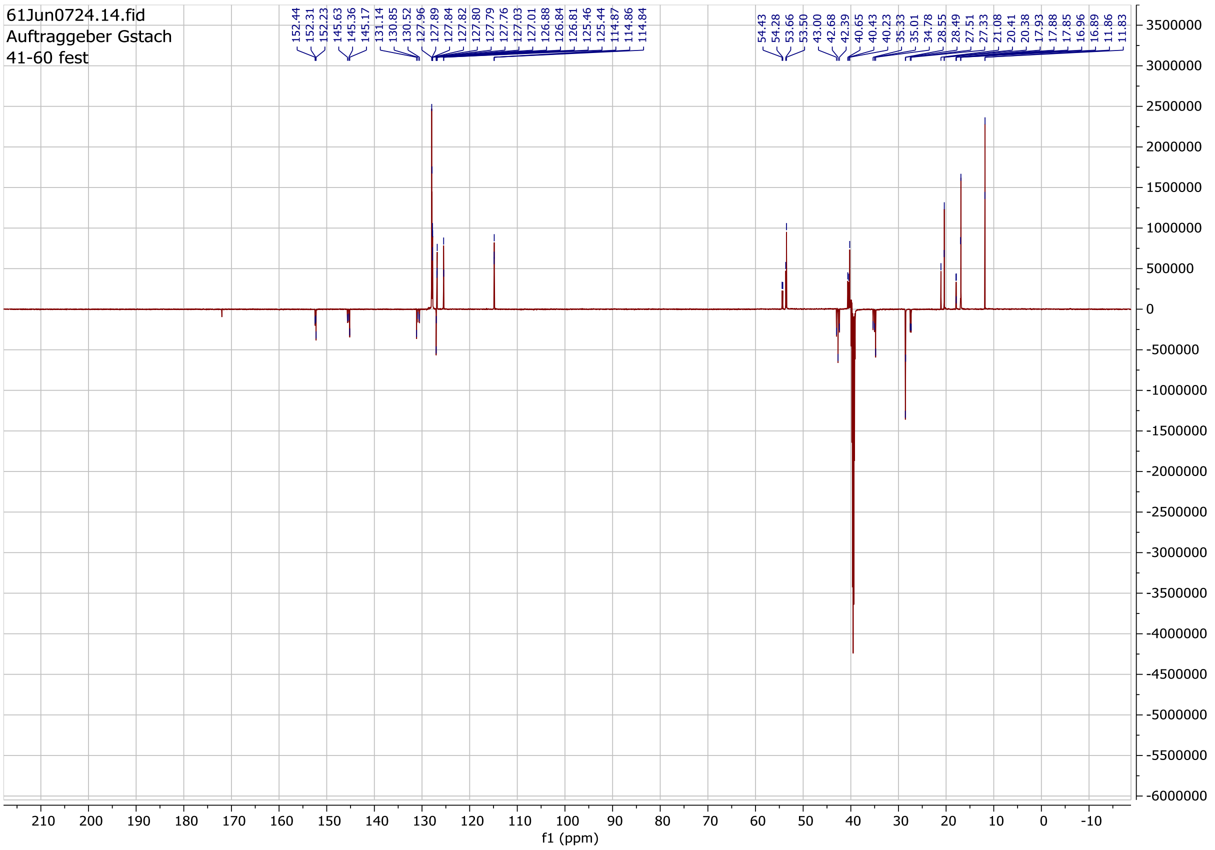
61Jun0724.14.fid
Auftraggeber Gstach
41-60 fest
152.44 152.31 152.23 145.63 145.36 145.17 131.14 130.85 130.52 127.96 127.89 127.84 127.82 127.80 127.79 127.76 127.03 127.01 126.88 126.84 126.81 125.46 125.44 114.87 114.86 114.84
54.43 54.28 53.66 53.50 43.00 42.68 42.39 40.65 40.43 40.23 35.33 35.01 34.78 28.55 28.49 27.51 27.33 21.08 20.41 20.38 17.93 17.88 17.85 16.96 16.89 11.86 11.83
f1 (ppm)
